# Social Anxiety, Cannabis Use Motives, and Social Context’s Impact on Willingness to Use Cannabis

**DOI:** 10.3390/ijerph18094882

**Published:** 2021-05-04

**Authors:** Elise Garrison, Conor Gilligan, Benjamin O. Ladd, Kristen G. Anderson

**Affiliations:** 1 Department of Psychology, Reed College, Portland, OR 97202, USA; eligarris@reed.edu; 2 School of Medicine and Public Health, College of Health, Medicine and Wellbeing, University of Newcastle, Callaghan 2308, Australia; Conor.Gilligan@newcastle.edu.au; 3 Department of Psychology, Washington State University Vancouver, Vancouver, WA 98642, USA; benjamin.ladd@wsu.edu

**Keywords:** cannabis, social anxiety, use motives, social contexts, emerging adults

## Abstract

Social anxiety is often purported to be a risk factor for increased cannabis use. Cannabis use motives are strong explanatory predictors of cannabis use embedded within social contexts. This investigation explored the impact of social anxiety, cannabis motives, and their interaction on willingness to use cannabis in a community sample of emerging adults. Social anxiety was anticipated to positively correlate with coping and conformity motives and greater willingness to use cannabis in peer social contexts. Motives to use were hypothesized to potentiate social anxiety’s influence on cannabis use decision-making. In total, 124 participants completed an audio simulation of social cannabis use contexts (Can-SIDE) and standard measures of social anxiety (SIAS) and use motives (MMM). Contrary to expectations, social anxiety exerted a protective effect on willingness to use cannabis, but only when conformity, social, and expansion motives were at or below average. These effects varied by social contexts of use. Social anxiety leading to increased cannabis use may be most apparent in clinical samples and in high-risk cannabis users, but this pattern was not supported in this sample of community living emerging adults below clinical cutoffs for cannabis use disorder with relatively high social anxiety.

## 1. Introduction

Cannabis use and cannabis-related consequences increase among emerging adults (18–25 year olds) compared to adolescents, and epidemiological data shows daily cannabis use among emerging adults is at the highest rate since the 1980s [1]. Social anxiety, or anxiety that an individual experiences in or in anticipation of social and/or performance situations, is associated with particularly high rates of problematic cannabis use [2]. In the U.S. National Comorbidity Survey, cannabis users with social anxiety were seven times more likely to experience serious cannabis-related impairment as compared to those who used cannabis without a concurrent social anxiety disorder [3]. In the U.S. in the past year, rates of social anxiety disorder were 15.5% among individuals with cannabis dependence compared to 7.1% in the general population, but only 5% among individuals with cannabis abuse [4,5]. Furthermore, some evidence suggests that rather than being a risk factor, social anxiety may serve as a protective factor against problematic substance use [6,7]. This suggests there may be important moderators that could help clarify the association between social anxiety and cannabis use, providing an understanding of the mechanisms by which social anxiety may predispose individuals to cannabis use and associated problems.

Some explanations for the mixed relationship between cannabis use and social anxiety involve motives, or reasons for using cannabis, particularly those around coping and conformity [2]. Based on Cox and Klinger’s (1988) motivational model [8], elucidated by Cooper in the 1990s, use motives are characterized along the dimensions of positive-negative reinforcement and internal-external focus [9]. Positive reinforcement cannabis motives include enhancement (internal: increase fun and enjoyment) and social context (external: social facilitation), while negative reinforcement motives are characterized by coping (internal: reduce negative affect) and conformity (external: reduce peer pressure). Expansion motives characterize enhancement of perceptual and cognitive experience and experiencing different states of consciousness [10], leading to debate as to where expansion lies in the classic two-dimensional model [11]. Enhancement and social motives are the most commonly endorsed motives for cannabis consumption with enhancement and coping motives often associated with increased frequency of use [11]. Using cannabis to cope with negative affect has been identified as a key predictor of cannabis-related problems [11,12], increased scores on the CUDIT-R, and DSM-5 symptoms [13]. Most research on cannabis use motives has focused on students, leaving open questions as to whether we can generalize findings from these two groups to a broader sample of emerging adults as well as other age groups [11].

Both coping and conformity motives for cannabis are relevant to social anxiety, as socially anxious individuals may be more vulnerable to using cannabis to regulate negative affective states (coping) and due to fear of social censure (conformity) [2]. Buckner and colleagues found that coping motives for cannabis use mediated the relation between social anxiety and cannabis use problems in a sample of undergraduate psychology students who reported using cannabis on average 1–2 times per month and that social anxiety was a significant predictor of coping and conformity motives for cannabis use. In response to a social stress task in the laboratory [14], current cannabis users (88.9% with cannabis use disorder) were most likely to endorse coping motives and enhancement motives; in a retrospective study of college students, social anxiety was associated with increased cannabis use specifically to reduce negative effects and increase positive effects in social contexts, but not to manage effects more generally [15]. 

Much of the literature on social anxiety, cannabis, and motives relies on aggregated self-reported data. While such data are not inherently problematic, this level of assessment may not capture important relationships at the event level. Given that the majority of cannabis use in emerging adults occurs in social situations [16,17,18], and that use motives are embedded within the contextual, or situational, characteristics of use [11], exploring the effect of social anxiety on cannabis use decision-making during social interactions is needed to shed further light on how social anxiety affects the development of problematic cannabis use. Realistic laboratory simulation methods allow for the modeling of willingness to consume substances in peer contexts for youth and predict self-reported substance use and consequences across time [19]. Using a video simulation of common social alcohol and cannabis use contexts for adolescents, Anderson et al. [20] examined behavioral willingness, or openness to engage in a given behavior when afforded the opportunity to do so [21], as a proxy for alcohol and cannabis use in the laboratory. Behavioral willingness for cannabis across situational contexts, or scenes, in the simulation was related to the frequency of current use, and the acceptance of control offers (non-alcoholic beverages/food designed to account for indiscriminate responding) within the simulation was inversely related to current alcohol and cannabis use. Longitudinal work with college students using an audio format [22] demonstrated that behavioral willingness on the simulation predicted potentially hazardous alcohol use (AUDIT) [23] 8 months later, above and beyond baseline, and a similar tobacco simulation used with university students in the Netherlands corresponded to tobacco use at baseline and 1 month later [24].

Across investigations, this methodology has demonstrated strong psychometric properties and affords the opportunity to examine behavioral willingness in aggregate across social contexts, and separately by peer characteristics (men vs. women) [25] and situational context (scene-level analysis) [24,26]. In a study focusing on alcohol use motives, coping motives for alcohol interacted with scene-level behavioral willingness at baseline to predict AUDIT scores 8 months later [26]. To date, these simulations of social context have not been applied to the study of social anxiety, motives, and cannabis use.

Notable gaps exist in the literature on social anxiety, motives, and cannabis use: (1) there is limited information on the impact of enhancement, social, and expansion motives in these models; (2) there is a lack of contextually-based assessment procedures for social cannabis use, the most common use contexts for emerging adults, and a highly salient impact on decision-making around cannabis for those with social anxiety; and (3) there is a limited examination of these factors in nonclinical community samples, which may miss some important components of the relationship between social anxiety and cannabis use, particularly any potential protective effects that may be observed in lower risk populations. As such, the aim of this study was to examine how social anxiety relates to behavioral willingness to use cannabis in simulated social contexts, while also investigating how all five motives (conformity, coping, enhancement, expansion, and social) may moderate this relation in a community (nonclinical) sample of emerging adults. The Cannabis–Simulated Intoxication Digital Elicitation (Can-SIDE) is an audio simulation procedure assessing willingness to accept peer offers to smoke cannabis under laboratory conditions in five common use contexts (scenes) for emerging adults (playing cards with two friends, small party, watching a televised event, large party, pre-loading before an event) based upon published procedures for alcohol and tobacco [22,24].

Using the Can-SIDE, we predicted that higher levels of social anxiety would positively correlate with behavioral willingness to accept offers to smoke cannabis. We anticipated that coping and conformity motives would positively relate to social anxiety and moderate relations between social anxiety and behavioral willingness to accept cannabis offers, such that higher motives to use would potentiate social anxiety’s influence on behavioral willingness. As less attention has been given to social, enhancement, and expansion motives in such models, we explored whether these motives function as a moderator of social anxiety’s influence on cannabis willingness. Given previous findings that expansion motives played a role in stress and anxiety in college students [27], we had reason to believe expansion motives might emerge as a moderator in these models.

## 2. Materials and Methods

### 2.1. Participants

The final sample for this study was 124 young adults (20–25 years old). Inclusion criteria included: being 18–25 years of age, being a U.S. resident, lifetime cannabis use, and scoring below the clinical cutoff of 12 on the CUDIT-R [28]. In order for participants’ data to be used in the analytic sample, they were required to complete 50% or more of the survey, score at least 80% on our attention checks, and provide a valid email address (for compensation purposes). The most common reasons for being excluded were failing the attention checks (*n* = 145) and exceeding the CUDIT-R cutoff (*n* = 221). Excluding those scoring above CUDIT-R cutoffs from the protocol provided ethical control to individuals with potential cannabis use disorders, by protecting them from exposure to substance-related cues when the investigators were unable to assess cravings in real time online, which is standard protocol when conducting these procedures within the laboratory [20,22].

### 2.2. Measures

#### 2.2.1. Demographics

Demographic questions determined age, gender identity (man, woman, non-binary, other), race (American Indian/Alaskan Native, Asian-American, Native Hawaiian/Native Pacific Islander, Black/African American, white/Caucasian, Multiracial, Other), ethnicity (Latinx, Non-Latinx), the highest level of education attained, and if participants had been diagnosed with social anxiety disorder (0/1). The race item was aggregated given the low endorsement of some identities within the sample.

#### 2.2.2. Attention Checks

Attention checks were included in this study to ensure that participants were reading and understanding the survey, in order to give accurate answers. An attention check question was included after each block in the survey, and participants had to score 80% or above on the attention checks as a whole in order to be eligible for inclusion in the analysis.

#### 2.2.3. CUDIT-R

The Cannabis Use Disorders Identification Test-Revised (CUDIT-R) [29], based upon the AUDIT [23], screened for possible cannabis use disorders (DSM-IV). This 9-item measure identifies if individuals have cannabis use problems, based on the past 6 months, with a maximum total score of 32. The cutoff for a cannabis use disorder is a score of 12. The sensitivity and specificity were 73% and 95%, respectively, for current cannabis use disorder [28]. The categories within the measure are based on consumption, cannabis problems (abuse), dependence, and psychological features. Cronbach’s alpha in this sample was 0.84.

#### 2.2.4. Can-SIDE

The Cannabis Simulation Intoxication Digital Elicitation (Can-SIDE) audio simulation examines willingness to accept peer offers to smoke cannabis under laboratory conditions, as smoking cannabis is the most frequent form of administration in the U.S. [30]. This simulation was tailored to young adults and consisted of five scenes presenting different use contexts (playing cards with two friends, small party, large party, watching sports with friends, and pre-loading (use before attending another event)). Participants listened to the simulation on a computer or smartphone with headphones and received cannabis offers or control offers (food or non-alcoholic beverages) during each of the five social situations. The participants were asked to pretend the actors in the scene were their friends, and after each offer, indicate their willingness to accept on a scale of 1 to 7, with higher scores indicating greater willingness. The simulation lasted about 30 min. To score this measure, the cannabis and control items were averaged separately for each of the five use contexts, and totals for both cannabis (α = 0.93) and control items (α = 0.86) were calculated to determine overall behavioral willingness (BW).

#### 2.2.5. Marijuana Motives Measure

The Marijuana Motives Measure (MMM) [10] was used to assess motives for cannabis use. It consisted of 25 items assessing enhancement (α = 0.65), coping (α = 0.53), social (α = 0.58), conformity (α = 0.62), and expansion motives (α = 0.78). Participants indicated on a 5-point scale the frequency in which they smoke cannabis for the reasons presented.

#### 2.2.6. Daily Sessions, Frequency, Age of Onset, and Quantity of Cannabis Use Inventory

We used the Daily Sessions, Frequency, Age of Onset, and Quantity of Cannabis Use Inventory (DFAQ-CU) [31] to assess frequency, age of onset, and quantity of cannabis consumed. This measure consists of 41 items, some of which are rated on scales, and some of which require narrative responses. In this study, 11 items measured frequency (α = 0.74) and 9 items measured the quantity of cannabis use (α = 0.90), with the remaining items used to screen and characterize the sample.

#### 2.2.7. Social Interaction Anxiety Scale

The Social Interaction Anxiety Scale (SIAS) [32] assessed social anxiety. This 20-item self-report scale measures distress when meeting and talking with others and is used in clinical and research settings on a scale of 0 to 4. Items are summed to produce a total measure score out of 80; a score of 43 or more indicates traditional social anxiety. This measure showed good reliability in this sample (α = 0.88).

### 2.3. Procedure

All study procedures were evaluated by the Reed College Institutional Review Board. Participants were recruited via Craigslist in two major metropolitan areas in the Pacific Northwestern United States and participated via Qualtrics. The survey began by screening for lifetime cannabis use, followed by the CUDIT-R, next showing the Can-SIDE with scenarios in randomized order, and finishing with the self-report survey measures in randomized order. We compensated participants with a $10 Amazon gift card if they met all inclusion criteria.

### 2.4. Analytic Plan

All analyses were conducted in Stata 16.0 (Stata LLC, 2020). We used multiple imputation to account for the 4% of missing data on the variables of interest, considered missing completely at random. Estimates were aggregated across M = 500 replications of the dataset using chained equations (*mi estimate: regress*). We examined model stability using Monte Carlo error estimates (*mcerror*), demonstrating that 500 replications were sufficient. When interactions were statistically significant, we estimated the significance of the simple slopes using the *mimrgns* command. Using the Johnson–Neyman method [33], we plotted the regions of significance in Excel for the statistically significant interactions to better describe the conditional effects of social anxiety on BW as a function of motive type. Bonferroni corrected *p*-values accounted for familywise error rate tests in the analysis.

## 3. Results

As shown in Table 1, the sample was between the ages of 20–25 (despite seeking/inviting 18–25-year-old adults), mostly white, and predominantly identified as male. The participants’ highest level of education was a graduate degree and about a third of the sample had a bachelor’s degree. The vast majority of the participants were not college students. A subset of the sample reported a diagnosis of social anxiety disorder (11.29%). Based on the inclusion criteria, the sample CUDIT-R score was below the cutoff for potential cannabis use disorders (48% of all potential participants screened for the study were excluded for reporting a score of 12 or higher). While below the clinical cutoff for social anxiety on average, our sample reported greater social anxiety (*M* = 34.52, *SD* = 11.38) than another community sample of 18–30-year-old adults (*n* = 286; *M* = 18.40, *SD* = 11.46), *t*(117) = 15.38, *p* < 0.00001, but below that of the clinical sample (*n* = 132; *M* = 43.93, *SD* = 11.84), *t*(117) = −8.99, *p* < 0.00001, as reported by Rodebaugh et al. [34]. The mean BW for cannabis was slightly above the midpoint of the scale (out of 7) (*M* = 4.24, *SD* = 1.07).

To validate the Can-SIDE in this sample, we correlated BW for cannabis with the quantity and frequency of cannabis use on the DFAQ, applying a Bonferroni corrected *p* value of 0.005. BW for cannabis in scenes 1 (card game), 2 (small party), 3 (large party), and 5 (pre-loading) was positively correlated with recent cannabis quantity, *r* = 0.26–0.56, *p* = 0.004–0.0001; scene 4 (watching sports on tv) did not correlate with cannabis quantity. However, BW for cannabis ratings for all scenes positively correlated with cannabis frequency, *r* = 0.27 to 0.42, *p* = 0.004 to <0.00001. At the bivariate level, SIAS positively correlated with conformity motives only, *r* = 0.25, *p* = 0.009 (Bonferroni *p* = 0.01), partially supporting our hypotheses.

To test moderation, we examined the interaction of SIAS and each motive separately in the prediction of BW for cannabis for each scene with demographic covariates and BW for control offers as covariates (Table 2 and Table 3). Bonferroni corrected *p*-values indicated that all regressions were statistically significant (*p* < 0.002). Given the primary effect of interest was the interaction term, we have only provided specific results for models with a significant interaction effect. Across the set of mi regressions, demographic covariates varied in their impact on BW for cannabis. Most frequently, racially minoritized groups had lower BW, and college students had higher BW for cannabis; while not statistically significant in all models, Latinx identity was most commonly associated with lower BW for cannabis if it was statistically significant (all estimates available upon request). BW control was a strong and positive predictor of BW for cannabis across regressions as expected and adequately served as a control for response styles within participants.

**Figure 1:**
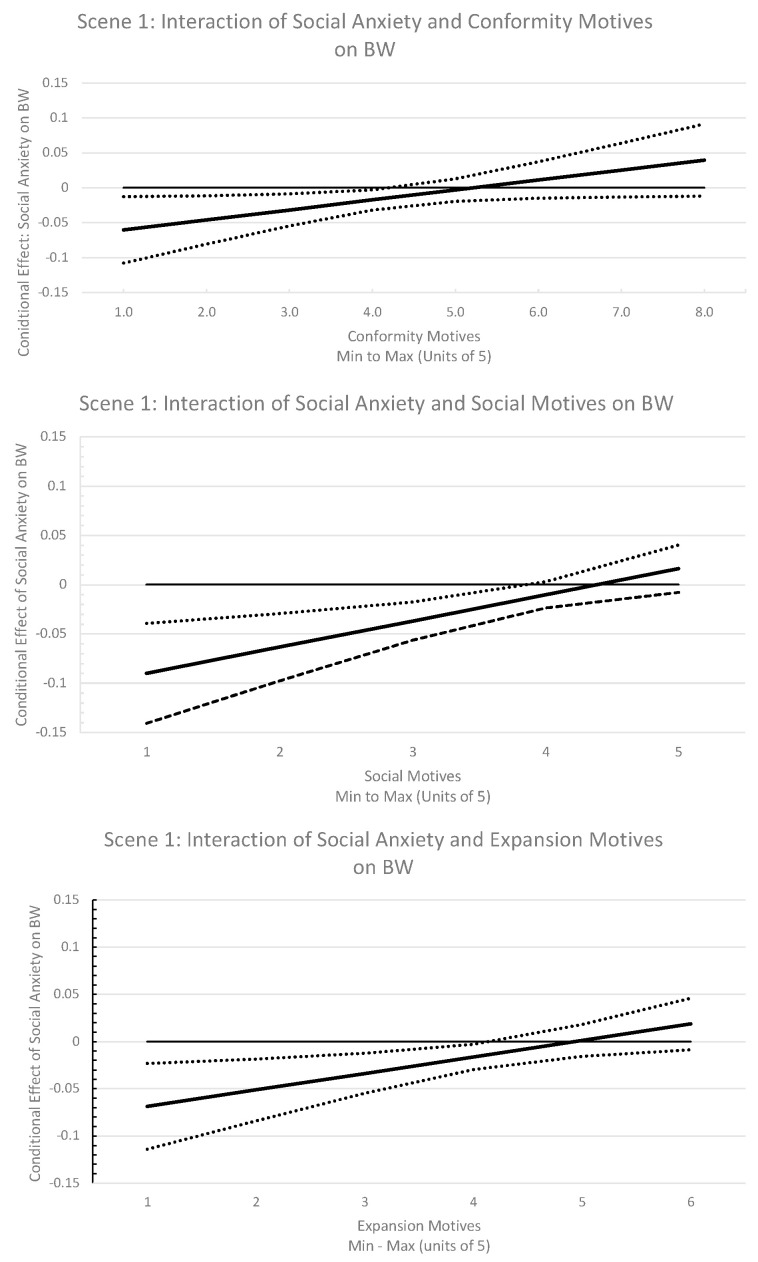
Visualizations of interactions in scene 1 (card game). Solid lines indicate the conditional effect of social anxiety on behavioral willingness at unit levels of cannabis use motives, ranging from the minimum to maximum values. Dotted lines represent the 95% confidence interval around these estimates.

Contrary to hypothesis, the main effect of SIAS was not a statistically significant predictor of BW for cannabis across scenes (tested independently and with the moderator). However, conformity motives, social motives, and expansion motives moderated the impact of social anxiety on BW for cannabis in the scene with two friends playing cards (Table 2), while coping and enhancement motives did not. Examination of the regions of influence suggested that social anxiety stopped demonstrating a unique effect on BW at greater than average levels of conformity motives. An almost identical pattern was found for social and expansion motives, whereby SIAS only demonstrated significant negative effects on BW for cannabis with below average levels of motives to use (Figure 1). As shown in Table 3, a similar pattern emerged such that SIAS had an inhibiting effect on BW for cannabis in the small party scene (five actors) when expansion motives were low, but had no impact when motives were at or above average. In the pre-loading scene (four actors), SIAS’ moderating effect only limited BW for cannabis when social motives were well below average.

## 4. Discussion

The purpose of this investigation was to explore the impact of social anxiety, cannabis motives, and their interaction on willingness to use cannabis in a community sample of emerging adults, who were subclinical cannabis users from the general population. Contrary to our hypotheses, this research uncovered a unique pattern of relationships between cannabis use willingness (BW), motives, and social anxiety. While we predicted that higher levels of social anxiety would result in greater BW, in fact, the opposite was true at low levels of conformity, social, and expansion motives. That is, at lower than average levels of motivation to use, there was a negative association between social anxiety and BW to use. When motives were at or above average, social anxiety had no impact on BW. These effects were only salient in the card playing, pre-loading, and small party scenes; the interactions were not demonstrated in the large party or watching a televised sporting event.

This unexpected pattern may be the product of a unique relationship occurring in this population group. With previous work in this space having focused on college students, and having included a broader range of use levels, it is possible that in a more general population of young adults who use cannabis at lower risk levels, subclinical social anxiety is in fact protective against use. Some research has suggested nondiagnostic social anxiety is negatively associated with cannabis use in young people [6,7]. One explanation for this inverse relationship is that a lower level of peer involvement mediates the relationship between social anxiety and cannabis use [35]. However, the current findings suggest that social anxiety also may serve to reduce willingness to use cannabis when in social situations, at least when use motives are low. While acknowledging that the limitations of the current study (e.g., cross-sectional design) prevent causal inferences from being drawn, these findings offer a possible pattern of association between social anxiety and cannabis use in an older, subclinical population, compared to those previously examined. This sample is older than the mean age of onset for social anxiety disorder of 15 years [36]. The risk of developing cannabis dependence is greater for those who begin using at age 17 and younger compared to individuals who began using cannabis at age 21 or older [37]. The mean age of cannabis use onset in the current sample was 18–19 years, suggesting a more stable group of lower risk young adults whose subclinical social anxiety may have protected them from using cannabis in social situations, possibly inhibiting progression to more problematic cannabis use.

This process could also explain the low levels of coping motives reported in this sample, as such motives may not be as relevant as in other samples where the more severe end of the cannabis use spectrum was not excluded. For example, in one study, social anxiety was seen to be robustly related to experiential avoidance (unwillingness to experience distressing internal states), which was in turn robustly related to coping motives [38]. That study’s participants were mostly heavy cannabis users, with 76% using daily. The observed relationship suggests that in the current sample reporting lower cannabis risk, individuals may be inclined to titrate their use in circumstances where they expect to be anxious. Furthermore, as the mean SIAS for the sample was lower than the clinical cutoff scores, one can also consider this sample subclinical for social anxiety. Thus, in this sample, even those who were more socially anxious remained below the threshold at which they may feel the need to use cannabis to cope. Although Buckner’s study on coping motives illuminates one potential pathway between social anxiety and cannabis use, this was not supported in our sample of nonclinical users who were also subclinical for social anxiety, suggesting that use pattern differences should be accounted for in future studies. Additionally, Buckner’s study examined self-reported cannabis use retrospectively, and ours specifically examined behavioral willingness to use cannabis in an audio simulation context.

While we did not find coping motives to be associated with social anxiety or behavioral willingness to use cannabis, conformity, social, and expansion motives did moderate the relation between social anxiety and behavioral willingness in at least one of the five use contexts. The different pattern of associations between scenes provides a case for further work to understand the context of use. Most previous research has relied on retrospective self-reported recall, but there is evidence to suggest that motive patterns may differ between contextualized and diary-based assessments. Further, previous work demonstrates that proximal aspects of decision-making are a product of relationships between motives and the environment, supporting the need to better understand the contextual/environmental factors driving differences in motivation and BW [8].

The idea lower risk users may be more discriminating in their decisions to use cannabis also is supported by the varied findings by social setting. The card-playing scene, with fewer individuals present, may represent a potentially less socially threatening scene than the large group party, and therefore, a safer setting in which to use cannabis. In the alcohol literature, one study that examined differences in context-specific alcohol outcome expectancies by social anxiety studied convivial, negative coping, and personal-intimate contexts [39]. It was found that high social anxiety was related to greater alcohol consumption because of tension reduction motives in negative coping and personal-intimate contexts. Interestingly, neither the watching sports on TV scene, a smaller social setting, nor the large party scene yielded significant associations with social anxiety and/or motives. Perhaps these represent the extremes of social situations in terms of stimulation, and such relationships only emerge when there is moderate social interaction/perceived threat.

A strength of this study was that the sample was less homogenous than previous studies; it was a community sample with participants between 20 and 25 years of age, with good representation of African-American, Latinx, and educational attainment. Another strength of this study was the inclusion of a comprehensive motives measure (MMM), allowing for analyses related to several different domains of cannabis use motives. It must be noted that the alpha levels for two of the four motive domains, coping (α = 0.53) and social (α = 0.58), indicated poor reliability, and therefore represent a limitation of the study. Corresponding alphas in previous studies have suggested better reliability, questioning the patterns of motivation among this lower-level user group. Participants who indicated a potential cannabis use disorder on the CUDIT-R were excluded from analyses; this truncation of the use spectrum may have led to important effects being missed compared to previous research including heavy users. However, the exclusion of these heavy users enabled this study to uncover other alternate patterns in cannabis use motives.

## 5. Conclusions

These results show that coping-motivated use was not commonly endorsed in this sample of subclinical cannabis users. It is likely that coping-motivated use is more associated with problem use, and therefore, represents a useful target for treatment-focused interventions. The present findings, however, suggest that other motives and the context of use are likely to be more appropriate targets for prevention and harm-minimization efforts in subclinical cannabis users. Like most samples of youth, our sample highly endorsed motives to use for fun, enjoyment (enhancement), and being with friends (social). Prevention efforts should address these motives directly, particularly as it relates to potentially high-risk cannabis use contexts. Given the focus on coping-motivated use in many prevention programs, addressing cannabis use in convivial situations with friends is also needed.

In the future, research on social anxiety, cannabis use motives, and BW could include participants with cannabis use disorder (adopting existing ethical approaches to mitigate their craving in the lab) to directly compare patterns among different levels of use. Data analysis that includes the full spectrum of users might reveal more information about motives, outside of those related to conformity. Future research should include participant interviews to explore differences in motives and willingness across scenes. More research in subclinical populations may point to possible explanations for how social anxiety is associated with willingness to use cannabis, including greater understanding of protective pathways, and provide guidance for the development of more effective treatment and prevention for young adults.

## Figures and Tables

**Table 1:** Demographics and sample characteristics (Total *N* = 124).

		*M* (*SD*)	*n* (%)
Age (*n* = 124)	Range: 20–25	23.13(1.51)	
Race (*n* = 124)	African American		19 (15.32)
	White		80 (64.52)
	Other		25 (20.16)
Ethnicity (*n* = 116)	Latinx		21 (18.10)
	Non-Latinx		95 (81.90)
Gender (*n* = 124)	Women		34 (27.42)
	Men		87 (70.16)
	Non-binary		3 (2.42)
Transgender (*n* = 123)	Yes		1 (0.81)
	No		122 (99.19)
College status (*n* = 124)	Students		24 (19.35)
	Non-students		100 (80.65)
Highest level of education (*n* = 124)	High school		25 (20.16)
	Some college		24 (19.35)
	Associates/Technical		33 (26.61)
	Bachelor’s degree or higher		42 (33.87)
Social anxiety diagnosis (*n* = 124)	Yes		14 (11.29)
	No		110 (88.71)
SIAS	Total score	34.52 (11.38)	
CUDIT-R	Total score	8.33 (1.87)	
DFAQ	Marijuana frequency	44.15 (15.16)	
	Marijuana quantity	9.96 (4.22)	
	Age of onset	18.38 (3.68)	

Note: *SIAS*: Social Interaction Anxiety Scale; *CUDIT-R*: Cannabis Use Disorder Identification Test-Revised; *FAQ*: Daily Sessions, Frequency, Age of Onset, and Quantity of Cannabis Use Inventory.

**Table 2:** Regression of behavioral willingness for cannabis on social anxiety, cannabis use motives, and interaction terms in the card game scene (2 actors) when interactions were statistically significant (*N* = 124).

	*B*	*SE*	*p*	*CI*
BW Control	0.36	0.07	<0.001	[0.22 to 0.51]
Frequency	0.05	0.01	<0.001	[0.02 to 0.08]
SIAS	−0.01	0.01	0.037	[−0.03 to −0.00]
Conformity	−0.03	0.02	0.244	[−0.07 to 0.02]
SIAS x Conf	0.00	0.00	0.041	[0.00 to 0.01]
Overall Model	*F* (9111.9) = 13.97, *p* < 0.00001			
BW Control	0.30	0.08	<0.001	[0.14 to 0.44]
Frequency	0.08	0.01	<0.001	[0.02 to 0.07]
SIAS	−0.02	0.01	0.003	[−0.03 to −0.01]
Social	0.04	0.00	0.170	[−0.02 to 0.09]
SIAS x Social	0.00	0.00	0.003	[0.00 to 0.01]
Overall Model	*F* (9111.9) = 14.48, *p* < 0.00001			
BW Control	0.40	0.08	<0.001	[0.25 to 0.55]
Frequency	0.04	0.01	<0.001	[0.02 to 0.07]
SIAS	−0.01	0.01	0.058	[−0.03 to −0.00]
Expansion	−0.02	0.02	0.511	[−0.06 to 0.03]
SIAS x Exp	0.00	0.00	0.012	[0.00 to 0.01]
Overall Model	*F* (9111.9) = 13.97, *p* < 0.00001			

Note: BW = behavioral willingness; Frequency = cannabis use frequency; Conformity or Conf = MMM conformity motives; Expansion or Exp = MMM expansion motives; Social = MMM social motives; all predictors were centered prior to analysis. Demographic covariates (race, ethnicity, college attendance) were included in all regressions; estimates available upon request. Missing data were accounted for using multiple imputation using chained equations (*M* = 500).

**Table 3:** Regression of behavioral willingness for cannabis on social anxiety, cannabis use motives, and interaction terms in the small party and pre-loading scenes when interactions were statistically significant.

Small Party/5 Actors	*B*	*SE*	*p*	*CI*
BW Control	0.40	0.08	<0.001	[0.22 to 0.56]
Frequency	0.03	0.01	0.058	[−0.00 to 0.06]
SIAS	−0.01	0.01	0.125	[−0.03 to −0.00]
Expansion	−0.00	0.03	0.990	[−0.05 to 0.05]
SIAS x Exp	0.00	0.00	0.027	[0.00 to 0.01]
Overall Model	*F* (9112) = 14.91, *p* < 0.00001			
Pre-loading/4 Actors				
BW Control	0.25	0.11	0.027	[0.03 to 0.48]
Frequency	0.01	0.02	0.479	[−0.02 to 0.04]
SIAS	−0.01	0.01	0.242	[−0.03 to 0.01]
Social	0.05	0.03	0.138	[−0.02 to 0.12]
SIAS x Social	0.00	0.00	0.049	[0.00 to 0.01]
Overall Model	*F* (9108) = 7.48, *p* < 0.00001			

Note: BW = behavioral willingness; Frequency = cannabis use frequency; Expansion or Exp = MMM expansion motives; Social = MMM social motives; all predictors were centered prior to analysis. Demographic covariates (race, ethnicity, college attendance) were included in all regressions; estimates available upon request. Missing data were accounted for using multiple imputation using chained equations (*M* = 500).

## Data Availability

The data can be accessed at https://www.reed.edu/psychology/ahrp/ (accessed on 12 March 2021).
